# Intra-adrenal murine TH-MYCN neuroblastoma tumors grow more aggressive and exhibit a distinct tumor microenvironment relative to their subcutaneous equivalents

**DOI:** 10.1007/s00262-015-1663-y

**Published:** 2015-02-17

**Authors:** Michiel Kroesen, Ingrid C. Brok, Daphne Reijnen, Maaike A. van Hout-Kuijer, Ingrid S. Zeelenberg, Martijn H. Den Brok, Peter M. Hoogerbrugge, Gosse J. Adema

**Affiliations:** 1 Department of Tumor Immunology, Radboud Institute for Molecular Life Sciences/278 TIL, Radboud University Medical Center, Post Box 9101, 6500 HB Nijmegen, The Netherlands; 2 Central Animal Laboratory, Radboud University Medical Center, Nijmegen, The Netherlands; 3 Present Address: Institute of Applied Sciences, HAN University of Applied Sciences, Nijmegen, The Netherlands; 4 Department of Pediatric Oncology, Radboud University Medical Centre, Nijmegen, The Netherlands; 5 Princes Máxima Center for Pediatric Oncology, De Bilt, The Netherlands

**Keywords:** Neuroblastoma, Orthotopic, Mouse model, Bioluminescence, Immunotherapy

## Abstract

In around half of the patients with neuroblastoma (NBL), the primary tumor is located in one of the adrenal glands. We have previously reported on a transplantable TH-MYCN model of subcutaneous (SC) growing NBL in *C57Bl/6* mice for immunological studies. In this report, we describe an orthotopic TH-MYCN transplantable model where the tumor cells were injected intra-adrenally (IA) by microsurgery. Strikingly, 9464D cells grew out much faster in IA tumors compared to the subcutis. Tumors were infiltrated by equal numbers of lymphocytes and myeloid cells. Within the myeloid cell population, however, tumor-infiltrating macrophages were more abundant in IA tumors compared to SC tumors and expressed lower levels of MHC class II, indicative of a more immunosuppressive phenotype. Using 9464D cells stably expressing firefly luciferase, enhanced IA tumor growth could be confirmed using bioluminescence. Collectively, these data show that the orthotopic IA localization of TH-MYCN cells impacts the NBL tumor microenvironment, resulting in a more stringent NBL model to study novel immunotherapeutic approaches for NBL.

**Electronic supplementary material:**

The online version of this article (doi:10.1007/s00262-015-1663-y) contains supplementary material, which is available to authorized users.

## Introduction

Neuroblastoma (NBL) is an aggressive malignancy of childhood with a dismal prognosis in high-risk patients. NBL arises from any of the parasympathetic nervous tissues in the body. In around half of patients, the primary tumor is located in one of the adrenal glands [1]. In a recent phase III trial, immunotherapy was added to the current standard therapy regimen, which consists of chemotherapy, surgery, radiation therapy and autologous stem cell transplantation followed by retinoic acid treatment [2]. The addition of this immunotherapy regimen to the standard treatment resulted in an improved survival of these high-risk NBL patients. However, since around half of these patients eventually did show progressive disease, a clear need remains to improve (immuno)therapy regimens for the treatment of NBL.

Most tumors are highly infiltrated by cells of the immune system exerting either pro- or anti-tumor effects, depending on cell type and activation status [3]. In established tumors, however, there is a state of chronic inflammation, paradoxically resulting in local immune suppression [4, 5]. Regulatory immune cell subsets are actively attracted to the tumor that interacts with each other and create an immunosuppressive microenvironment [6]. For example, M2 macrophages infiltrating tumors can actively suppress anti-tumor immune responses via the production of anti-inflammatory cytokines [7, 8]. In NBL tumors, the presence of tumor-associated macrophages was shown to be an independent prognostic factor associated with an adverse prognosis^8^. The immunosuppressive environment also contributes to progressive or recurrent disease during or after immunotherapy. Counteracting the tumor-induced immune suppression therefore is an important step in improving cancer immunotherapy [9]. To achieve this, there is a need for a more comprehensive understanding of the mechanisms involved in the process of tumor-induced immune suppression. To get a better understanding of the immunosuppressive environment of NBL tumors and to find novel ways to counteract the local immune suppression, relevant preclinical tumor models are needed [10].

Genomic amplification of the proto-oncogene MYCN is consistently associated with a poor prognosis and occurs in around 20 % of primary NBL tumors [11]. The TH-MYCN transgenic mouse model of NBL is driven by the over expression of MYCN in the developing sympathetic nervous system, eventually resulting in paraspinous NBL [12]. In our transplantable model, we inject the tumor cells into the adrenal gland, which is a different location than the tumors arising in the TH-MYCN transgenic, but more like in human adrenal NBL [13]. The spontaneous arising NBL tumors in the TH-MYCN model are very similar to high-risk human NBL with respect to tumor histology and genetics [14, 15]. Cell lines were derived from NBL tumors that arose in transgenic TH-MYCN mice. Transplantation of these cell lines in syngeneic mice also resulted in NBL tumors with similar histology and genetics compared to human NBL [16, 17]. We recently set up and described such a transplantable TH-MYCN model by transplanting the TH-MYCN-derived NBL cell line 9464D in C57Bl/6 mice [18]. In these studies, we found that the immunological properties of the transplantable TH-MYCN model were similar to those of high-risk human NBL, rendering this model a powerful tool in the preclinical development of novel immunotherapies for NBL [19].

In our previous studies, the TH-MYCN 9464D NBL cells were injected subcutaneously (SC), allowing for external measurement of tumor growth [18]. In this study, we established an orthotopic transplantable TH-MYCN model using 9464D NBL cells and a luciferase expressing 9464D variant. Interestingly, the orthotopic intra-adrenal (IA) NBL tumors grew out much faster compared to SC NBL tumors and were more heavily infiltrated by macrophages exhibiting an immunosuppressive M2 phenotype. We conclude that this orthotopic transplantable TH-MYCN model represents a highly relevant model to develop and understand the mechanisms of novel immunotherapies for NBL.

## Materials and methods

### Animals and cell lines

6- to 8-week-old female C57Bl/6J wild-type (WT) mice were purchased from Charles River. Animals were held under specified pathogen-free conditions in the Central Animal Laboratory (Nijmegen, the Netherlands). All experiments were performed according to the guidelines for animal care of the Nijmegen Animal Experiments Committee. The transgenic cell line 9464D was derived from spontaneous tumors from TH-MYCN transgenic mice on C57Bl/6 background and was a kind gift from Dr. Orentas (NIH, Bethesda). 9464D cells were cultured in Dulbecco’s modified Eagle medium (DMEM) containing 10 % fetal calf serum (FCS), 1 % non-essential amino acids, 1.0 % amino acids (AA) and 0.05 % β-mercaptoethanol (medium). 9464D-luc cells were generated as described below and cultured in medium containing G418 (200 ng/ml).

### Generation of 9464D-luciferase (9464D-luc) clone

9464D cells were transfected with a construct containing a green fluorescent protein (GFP)-Ires-Luciferase sequence under the control of a cytomegalovirus (CMV) promoter. Stably transfected 9464D cells were selected by adding increasing amounts of G418 to the medium to a final concentration of 200 ng/ml. GFP-positive cells were FACsorted and plated as single cells in 96-well plates. GFP-positive clones were expanded and selected for stable transfection by monitoring GFP-positivity by FACS. Luciferase expression was determined using a luciferase reporter assay; 1 × 10^5^ cells were lysed in 100 μl Passive Lysis Buffer (Promega), and the lysates were analyzed for luminescence with the use of the Dual-luciferase Reporter Assay System (Promega, Leiden, the Netherlands) according to manufacturer’s protocol and a Victor 3 luminometer (PerkinElmer, Groningen, the Netherlands). Several stably transfected clones were expanded, tested for cell surface marker expression and frozen for later use.

### Subcutaneous and intra-adrenal injection of 9464D and 9464D-luc cells

To compare subcutaneous and intra-adrenal tumor growth, 1 × 10^6^ 9464D or 9464D-luc cells from the same batch were injected either subcutaneously or intra-adrenally. Viability of tumor cells before injection was determined by tryptan blue staining and always exceeded 95 %. For intra-adrenal injections, microsurgery was performed by a skilled biotechnician. Institutional protocols and guidelines were adhered to for the delivery of anesthesia and analgesia. Briefly, a dorsal incision was made right lateral to the spinal cord, and the retroperitoneum was accessed. The kidney and adrenal gland were located, and using a 0.3-ml Becton Dickinson (BD) Micro-Fine™ needle, 1 × 10^6^ 9464D or 9464D-luc cells were injected in a volume of 30 μl into the adrenal gland. Using sutures, the retroperitoneum and skin were closed. Within 7 days, sutures could be removed and wounds were fully healed without any signs of inflammation.

### Monitoring tumor growth by bioluminescence

Tumor growth of 9464D-luc tumors at subcutaneous and adrenal sites was monitored in time. To prevent blockage of light by the black fur, the mice were shaved before each measurement. Mice were injected intraperitoneally with 150 μg D-Luciferin (PerkinElmer, Massachusetts, USA) in 200 μl phosphate buffered saline (PBS) and anaesthetized using isoflurane. Ten minutes after injection of D-Luciferin, mice were imaged using an in vivo imaging system (IVIS) Lumina (Xenogen, Almeda, CA, USA) camera by taking consecutive 10-s to 2-min imaging frames.

### Generation of single cell suspensions of tumors

Tumors were excised on indicated time points and mechanically dissociated using large needles followed by enzymatic digestion with 1 mg/ml collagenase type III (Worthington) and 30 μg/ml DNase type I (Roche) for 1 h at 37 °C. After 1 h, ethylenediaminetetraacetic acid (EDTA) was added (1.5 mM final concentration) and single cell suspensions were made by passing the tumor fragments over 100-μm cell strainers.

### Antibodies and flow cytometry

For flow cytometric analysis, the directly labeled mAbs used were CD11b-PerCP (M1/70), CD4-APC-Cy7 (L3T4) and Ly-6G-PE-Cy7 (1A8) from Biolegend, CD45.2-FITC (104), CD11c-APC (HL3), NK1.1-PE (PK136), Ly-6C-APC-Cy7 (AL-21) and CD3-PE (145-2C11) from BD, MHC II-PE (M5/114.15.2) from eBioscience. Cells for staining were washed in PBS and incubated with Fixable Viability Dye eFluor 780 (eBioscience). Cells were resuspended in ice-cold PBA and transferred to a V-bottom 96-wells plate. After incubation for 30 min with PBA containing anti-CD16/CD32 Fc-block (BD), cells were stained using specific monoclonal antibodies or the appropriate isotype controls for a period of 30 min. Cells were washed in PBA and measured on a Cyan apparatus (BD) and analyzed using Summit software.

### Statistics

Data were analyzed using GraphPad 5.0 software. Data are presented as means ± standard error of the mean (SEM). An unpaired *T* test (confidence intervals 95 %) was used to determine significant differences between two groups. Correlation between tumor weight and bioluminescent signal was determined using a Pearson correlation test (confidence intervals 95 %).

## Results

### 9464D tumors grow out faster in the adrenal gland compared to the subcutis

**Fig. 1:**
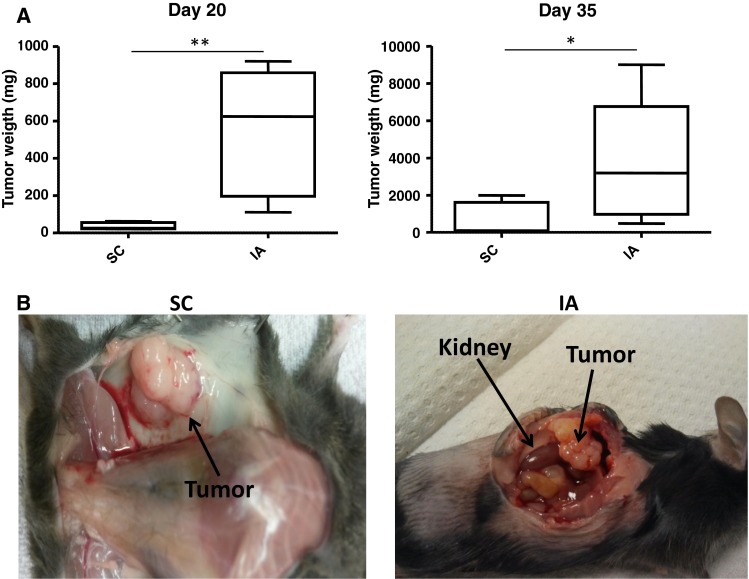
9464D NBL tumors grow faster in the adrenal gland compared to the subcutis. **a** Tumor weights of SC and IA 9464D tumors harvested on day 20 and day 35 were compared (*p < 0.05; **p < 0.01). **b** Tumors grow out and destruct the adrenal gland as shown by necropsy

We determined tumor growth of 9464D NBL cells at SC and IA sites. A significant difference in tumor weight between SC and IA tumors was readily observed at day 20 post-inoculation (Fig. 1a). Isolation of tumors at day 35 revealed that IA tumors were again significantly larger compared to SC tumors (Fig. 1a). IA tumors grew out aggressively, thereby completely destructing the adrenal gland, as was evident at necropsy (Fig. 1b). Similar to human NBL and to previous reports of TH-MYCN transgenic tumor models [17, 20, 21], the 9464D tumors were hypervascular already on macroscopic examination (Fig. 1b). These data show that 9464D tumors grow out faster in the adrenal gland compared to the subcutis.

### Adrenal tumors are infiltrated by myeloid cells having a more immunosuppressive phenotype

**Fig. 2:**
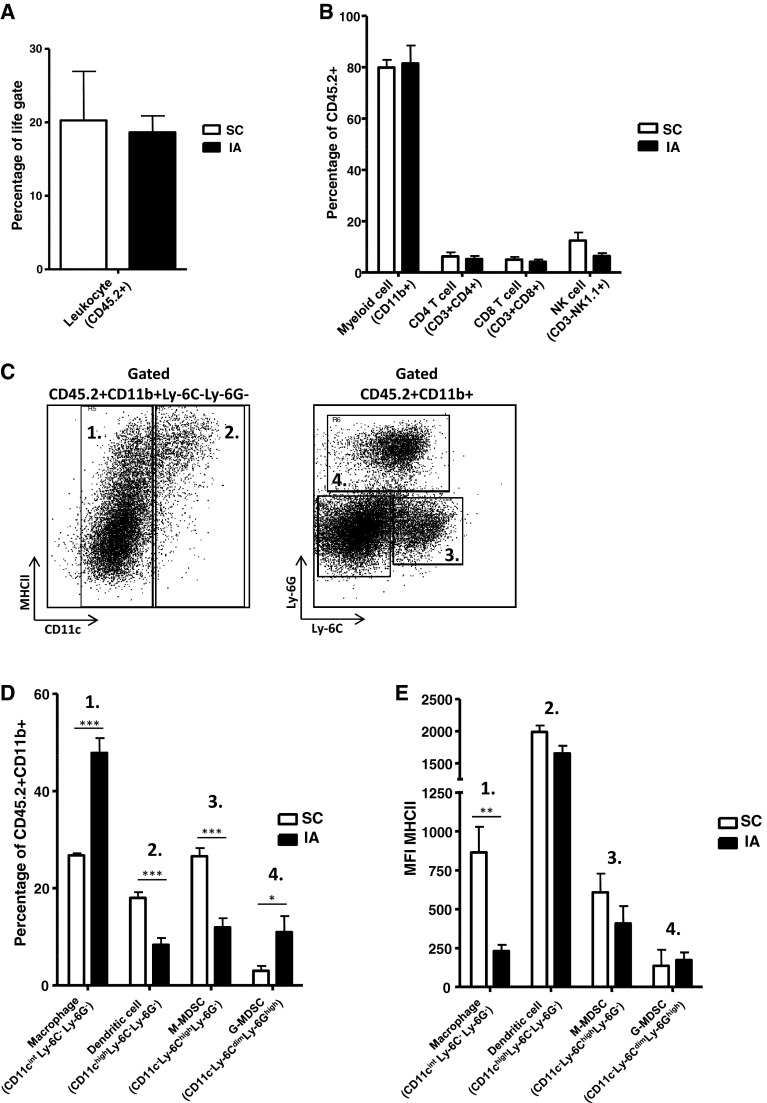
Intra-adrenal NBL tumors are highly infiltrated by macrophages expressing lower levels of MHCII. **a** Leukocyte infiltration is similar for SC and IA tumors. Percentages of CD45.2+ TIL of life-gated total tumor cells are depicted for SC and IA tumors. **b** The distribution of immune cell types within the TIL was similar for SC and IA tumors. CD45.2+ TIL were gated and analyzed for the expression of CD11b, CD3, CD4, CD8 and NK1.1. Percentages of CD11b+ myeloid cells, CD3+CD4+ and CD3+CD8+ T cells and CD3-NK1.1+ NK cells are depicted for SC and IA tumors. **c**, **d** The distribution of myeloid cell subsets within total CD11b+ myeloid cells is altered between SC and IA tumors. CD45.2+CD11b+ tumor-infiltrating myeloid cells were gated and analyzed for the expression of CD11c, Ly-6C, Ly-6G and MHCII. Percentages of CD11c^int^Ly-6C^−^Ly-6G^−^ macrophages, CD11c^high^Ly-6C^−^Ly-6G^−^ dendritic cells, CD11c^−^Ly-6C^high^Ly-6G^−^ M-MDSC and CD11c^−^Ly-6C^dim^Ly-6G^high^ G-MDSC are depicted for SC and IA tumors. (***p < 0.001). **e** Macrophages expressing lower levels of MHCII in IA tumors compared to SC tumors. MFI’s of MHCII on previously gated myeloid cell subsets are depicted for SC and IA tumors (**p < 0.01)

Next, we compared immune infiltration of IA tumors harvested on day 20 with SC tumors harvested on day 35. Tumors harvested at these time points showed comparable tumor weight averages of 624.8 (±357.8 SEM) and 557.0 (±143.8 SEM) mg (*p* = 0.86), respectively. Tumors growing at both anatomical sites were equally infiltrated by CD45.2+ tumor-infiltrating leukocytes (TIL); the percentage of CD45.2+ TIL of the total tumor cell suspension was around 20 % for both anatomical locations (Fig. 2a). The amounts of CD11b+ myeloid cells, CD3+CD4+ and CD3+CD8+ T cells as well as CD3-NK1.1+ natural killer (NK) cells within the CD45.2+ TIL were also similar for SC and IA tumors (Fig. 2b). Similar to our previous observations [18], the largest population of tumor-infiltrating leukocytes at both tumor locations was CD11b+ myeloid cells, making up around 80 % of all CD45.2+ TIL (Fig. 2b). Using mAb directed toward the cell surface markers CD11c, Ly-6C, Ly-6G and MHC class II, we were able to identify some of the major myeloid cell subsets (Fig. 2c). We observed several alterations in the distribution of these myeloid cell populations (Fig. 2d). In IA tumors, CD11c^int^Ly-6C^−^Ly-6G^−^MHCII^int^ cells having a phenotype consistent with macrophages were significantly increased compared to SC tumors (Fig. 2d). The percentages of CD11c^high^Ly-6C^−^Ly-6G^−^MHCII^high^ dendritic cells (DC) were decreased in IA tumors compared to SC tumors. Also, CD11c^−^Ly-6C^high^Ly-6G^−^MHCII^low^ cells having a phenotype consistent with monocytic myeloid-derived suppressor cells (M-MDSC) were decreased in IA tumors compared to SC tumors. In contrast, the CD11c^−^Ly-6C^dim^Ly-6G^high^MHCII^low^ cells having a phenotype consistent with granulocytic myeloid-derived suppressor cells (G-MDSC) were increased in IA tumors compared to SC tumors (Fig. 2d). To further determine the phenotype of these tumor-infiltrating macrophages in SC and IA tumors, the surface expression of MHCII on these cells was determined (Fig. 2d). The expression levels of MHC class II on DC, M-MDSC and G-MDSC in IA and SC tumors, however, were not altered. In contrast, tumor-infiltrating macrophages in IA tumors expressed significantly lower levels of MHC class II compared to macrophages infiltrating SC tumors, suggesting a more M2 phenotype (Fig. 2e).

**Fig. 3:**
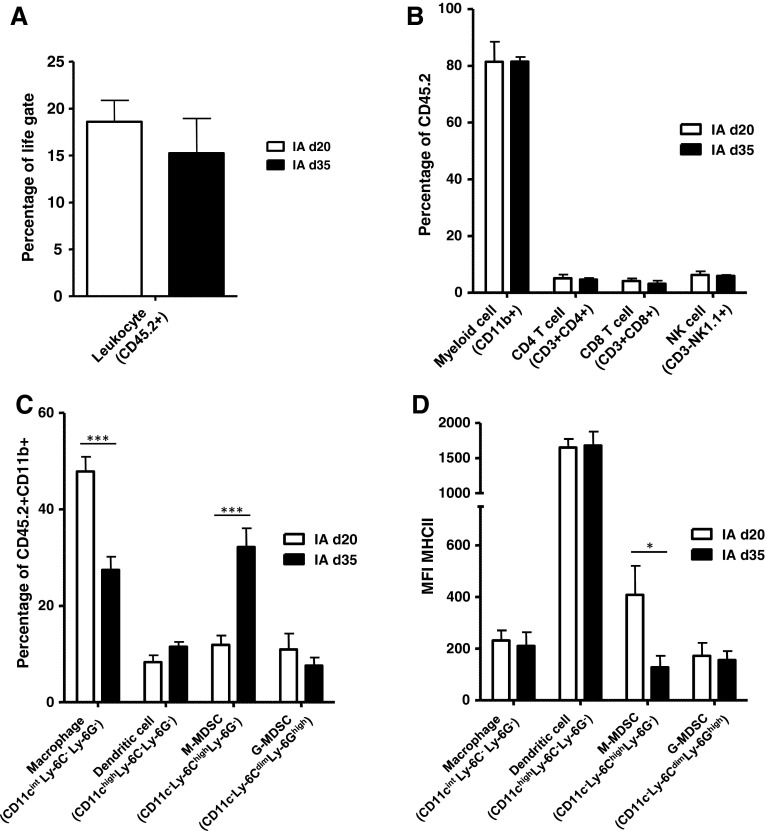
Late-stage adrenal tumors are highly infiltrated by M-MDSC expressing lower levels of MHCII. **a** Percentage of tumor-infiltrating leukocytes (TIL) is similar for day 20 (d20) and day 35 (d35) intra-adrenal tumors. Percentages of CD45.2+ TIL of life-gated cells are depicted for d20 and d35 IA tumors. **b** The distribution of immune cell types within the TIL was similar for d20 and d35 IA tumors. Percentages of CD11b+ myeloid cells, CD3+CD4+ and CD3+CD8+ T cells and CD3-NK1.1+ NK cells are depicted for d20 and d35 IA tumors. **c** The distribution of myeloid cell subset within total CD11b+ myeloid cells is altered between d20 and d35 IA tumors. CD45.2+CD11b+ tumor-infiltrating myeloid cells were gated and analyzed for the expression of CD11c, Ly-6C, Ly-6G and MHCII. Percentages of CD11c^int^Ly-6C^−^Ly-6G^−^ macrophages, CD11c^high^Ly-6C^−^Ly-6G^−^ dendritic cells, CD11c^−^Ly-6C^high^Ly-6G^−^ M-MDSC and CD11c^−^Ly-6C^dim^Ly-6G^high^ G-MDSC are depicted for d20 and d35 IA tumors (***p < 0.001). **d** M-MDSC in late-stage adrenal tumors expressing lower levels of MHCII. CD45.2+CD11b+ myeloid cells subsets were analyzed for the expression of MHCII. MFI’s of MHCII on these cell types is depicted for d20 and d35 IA tumors (*p < 0.05)

Upon analysis of IA NBL tumors harvested at day 35, these ‘late-stage’ IA tumors were equally infiltrated by CD45.2+ TIL, compared to the ‘early-stage’ day 20 IA tumors (Fig. 3a). Within the CD45.2+ TIL, infiltration of CD11b+ myeloid cells, CD3+CD4+ and CD3+CD8+ T cells and CD3-NK1.1+ NK cells were also similar between ‘late-stage’ and ‘early-stage’ IA tumors (Fig. 3b). Upon analysis of the CD45.2+CD11b+ myeloid cells, however, the presence of macrophages was reduced, while cells having a phenotype of M-MDSC were significantly increased in late-stage compared to early-stage IA tumors (Fig. 3c). Interestingly, while the tumor-infiltrating macrophages expressed similar low levels of MHC class II, the more abundant M-MDSC now also showed a significant reduction in the expression of MHC class II (Fig. 3d).

### Orthotopic 9464D tumors can be monitored semiquantitatively using bioluminescence

**Fig. 4:**
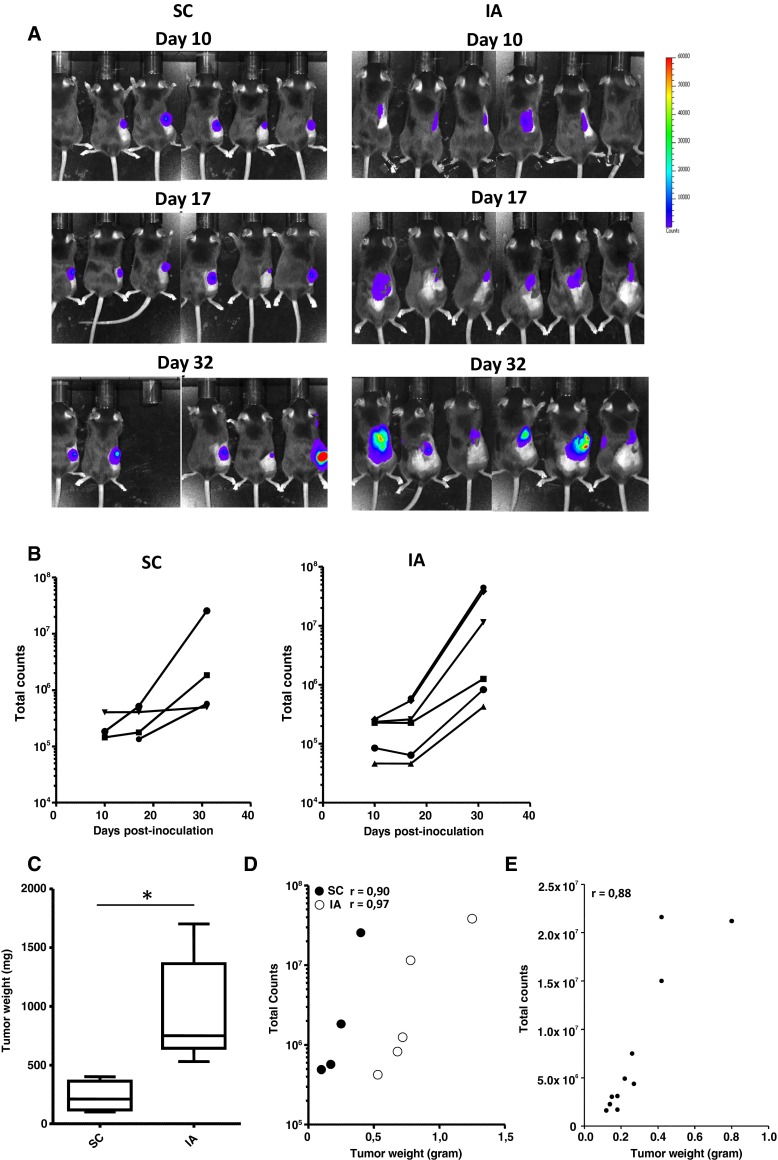
Orthotopic 9464D-luc tumors show similar enhanced tumor growth and can be monitored semiquantitatively using bioluminesence. **a**, **b** SC and IA 9464D-luc tumors were monitored in time using bioluminescence (six mice/group, two mice in the SC group were excluded from the analysis due to skin ulcerations). **c** On day 32 post-inoculation, tumor weights of SC and IA 9464D-luc were compared. **d** Tumor weight ex vivo significantly correlates with the bioluminescent signal in vivo (Pearson *r* = 0.90; *p* = 0.049 for SC tumors, Pearson *r* = 0.97; *p* = 0.0034 for IA tumors). **e** Adrenal tumor weight ex vivo significantly correlates with the bioluminescent signal in vivo in a separate experiment (11 mice) (Pearson *r* = 0.88; *p* = 0.003)

9464D-luc cells were generated, which were stably bioluminescent in luciferase assays and expressed equal levels of immune-related cell surface molecules MHC class I, retinoic acid early transcript 1 (Rae1), programmed death ligand 1 (PD-L1) and the tumor antigen GD2 in vitro compared to the parental 9464D cell line (Suppl. Fig. 1) [18]. Following an initial plateau phase, an exponential increase in bioluminescent signal was observed for both SC and IA 9464D-luc tumors in time (Fig. 4a, b). Similar to the parental 9464D tumors, we observed a significant growth difference between SC and IA 9464D-luc tumors (Fig. 4c). There was a significant correlation between tumor size and the corresponding bioluminescent signal, showing that semiquantitative measurement of tumor size was feasible for both SC and IA tumors (Fig. 4d). The correlation between NBL tumor weight and the bioluminescent signal for IA growing tumors was confirmed in an independent experiment (Fig. 4e). We observed around 15 % CD45.2+ TIL in IA 9464D-luc tumors, similar to parental 9464D tumors (Suppl. Fig. 2A and Fig. 2A). IA 9464D-luc tumors were also highly infiltrated by CD11b+ myeloid cells (Suppl. Fig. 2B). Within the tumor-infiltrating CD45.2+CD11b+ myeloid fraction, similar myeloid cell subset presence and distribution compared to the IA growing parental 9464D tumors was observed (Suppl. Fig. 2C).

In conclusion, IA 9464D NBL tumors have a distinct, more immunosuppressive tumor microenvironment and can be monitored using bioluminescence.

## Discussion

In this report, we describe an orthotopic model of NBL using TH-MYCN transgenic NBL cells. We observed that IA 9464D NBL tumors grew faster compared to SC tumors. IA NBL tumors were more heavily infiltrated by macrophages expressing low levels of MHC class II, suggesting a more immunosuppressive microenvironment. Using luciferase-transfected NBL cells, it was feasible to monitor tumor growth in the adrenal gland in a semiquantitative manner by measuring the bioluminescence.

In previous studies, orthotopic tumors were shown to resemble the tumor biology of human tumors more closely than ectopic tumors [22, 23]. The local tumor microenvironment can influence both tumor cell biology as well as tumor cell invasion or metastasis and even therapy responses [23–29]. For example, injection of syngeneic pancreatic adenocarcinoma cells in the pancreas resulted in enhanced tumor growth compared to subcutaneous sites and differential expression of the tumor antigen Muc1 [30]. Our data also show that orthotopic NBL tumors growing in the adrenal gland have a different growth pattern and immune infiltration compared to ectopic growing NBL tumors. A plausible explanation for the faster 9464D NBL growth at orthotopic sites is the induction of a more immunosuppressive environment. We observed marked alterations in myeloid cell subsets and phenotype between SC and IA tumors of similar size. The most striking finding was that IA tumors were more densely infiltrated with macrophages expressing low levels of MHC class II, suggesting a more M2-like macrophage phenotype. Asgharzadeh et al. [8] showed that the infiltration of M2-like macrophages in disseminated NBL tumors was associated with an adverse prognosis. Therefore, our data suggest that the local environment in IA NBL tumors is more immune suppressive, resulting in more aggressive tumor growth.

These data are in agreement with data from Carlson et al. [31], showing that tumor-infiltrating macrophages acquire a more M2 phenotype in spontaneous TH-MYCN transgenic tumors. Thus, the immune infiltration in our orthotopic transplantable TH-MYCN model shows high similarity with the immune infiltration observed in NBL tumors in the original TH-MYCN transgenic mouse. NBL tumors in these TH-MYCN transgenic mice, however, arise in a stochastic fashion after a latent period. Tumor growth can be controlled more easily using the transplantable TH-MYCN model presented here. This model therefore allows for faster screening of novel therapies for NBL.

In most orthotopic tumor models, as in our orthotopic TH-MYCN model, it was not feasible to monitor tumor growth via external measurements. For this purpose, we generated a stably transfected clone of 9464D cells expressing firefly luciferase. Using this 9464D-luc cell line, we were able to monitor growth of SC and IA tumors. Tumor size and bioluminescent signals significantly correlated, suggesting that semiquantitative monitoring of tumor growth was feasible. The observed correlation between tumor size and bioluminescent signal also suggests that the NBL tumors are not highly necrotic, as extensive tumor necrosis was shown to result in a loss of correlation between tumor size and bioluminescent signal [32]. The black fur of C57Bl/6 mice can significantly block the light coming from the tumor. Shaving of the skin at the imaged areas was therefore performed to prevent loss of signal. In SC tumors, the bioluminescent signal was higher compared to IA tumors, most likely as a result of the more superficial location of the subcutaneous tumors. One drawback of using bioluminescence in an immunological model is the introduction of potential novel tumor antigens. The 9464D-luc cells not only expressed firefly luciferase, but also GFP as a reporter protein. Both luciferase and GFP are foreign proteins in the mouse and can be recognized by the host immune system. This potentially increases the immunogenicity of the tumor cells resulting in a more immunogenic tumor model. Therefore, effective novel immune therapeutics in the 9646-luc model are preferentially also tested in the parental 9464D model and/or the spontaneous TH-MYCN transgenic mouse model.

In summary, IA 9464D NBL tumors grow faster and are infiltrated by myeloid cells having a more immunosuppressive phenotype compared to SC NBL tumors. The feasibility of using bioluminescence allows for careful monitoring of the effect of novel therapies on tumor growth. We conclude that this orthotopic transplantable TH-MYCN model is a relevant model to study multiple aspects of the immunobiology of NBL and to develop novel (immuno)therapeutics for NBL.

## Electronic supplementary material

Below is the link to the electronic supplementary material.

Supplementary material 1 (PDF 369 kb)

## Abbreviations

AA: Amino acids
BD: Becton Dickinson
CMV: Cytomegalovirus
DC: Dendritic cells
DMEM: Dulbecco’s modified Eagle medium
EDTA: Ethylenediaminetetraacetic acid
FCS: Fetal calf serum
GFP: Green fluorescent protein
G-MDSC: Granulocytic myeloid-derived suppressor cells
IA: Intra-adrenal
IVIS: In vivo imaging system
M-MDSC: Monocytic myeloid-derived suppressor cells
NBL: Neuroblastoma
NK cell: Natural killer cell
PBS: Phophate buffered saline
PD-L1: Programmed death ligand 1
Rae1: Retinoic acid early transcript 1
SC: Subcutaneous
SEM: Standard error of the mean
TIL: Tumor infiltration leukocytes
WT: Wild-type
