# Supplementary material for: Intra-adrenal murine TH-MYCN neuroblastoma tumors grow more aggressive and exhibit a distinct tumor microenvironment relative to their subcutaneous equivalents
Source: Cancer Immunol Immunother. 2015 Feb 17;64(5):563–72. doi: 10.1007/s00262-015-1663-y (PMC4412512; doi:10.1007/s00262-015-1663-y)
Supplement: Supplementary file 1 — Supplementary material 1 (PDF 369 kb) [file 262_2015_1663_MOESM1_ESM.pdf]

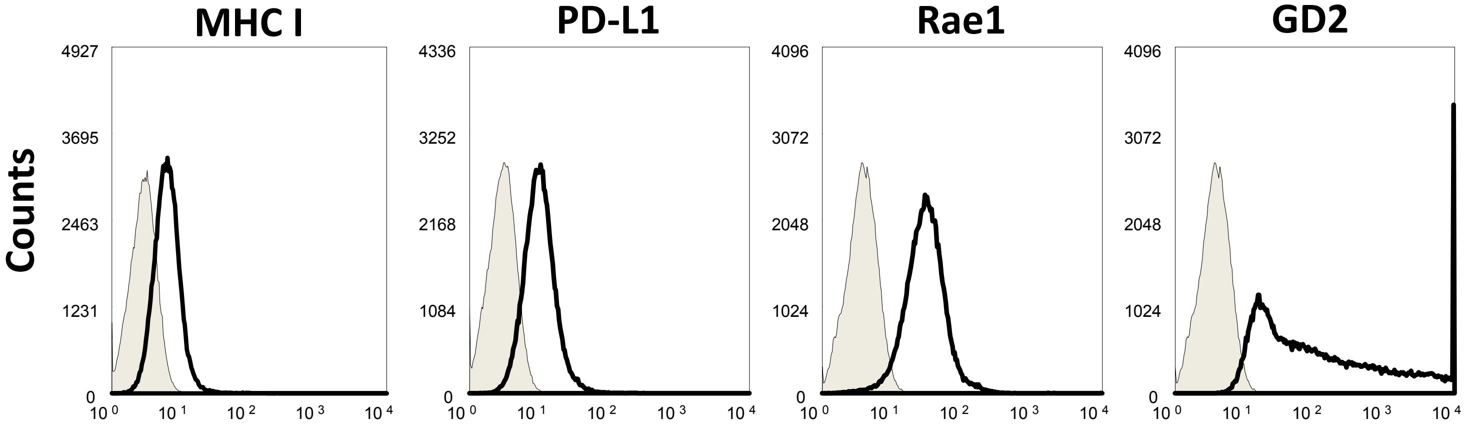

Supplementary Figure 1: 9464D-luc cells express relevant immune related surface molecules. 9464D-luc cells express low levels of MHCI and PD-L1 and highly express Rae1 and GD2 as determined by flow cytometry. Grey shading= isotype control, Black line= specific staining

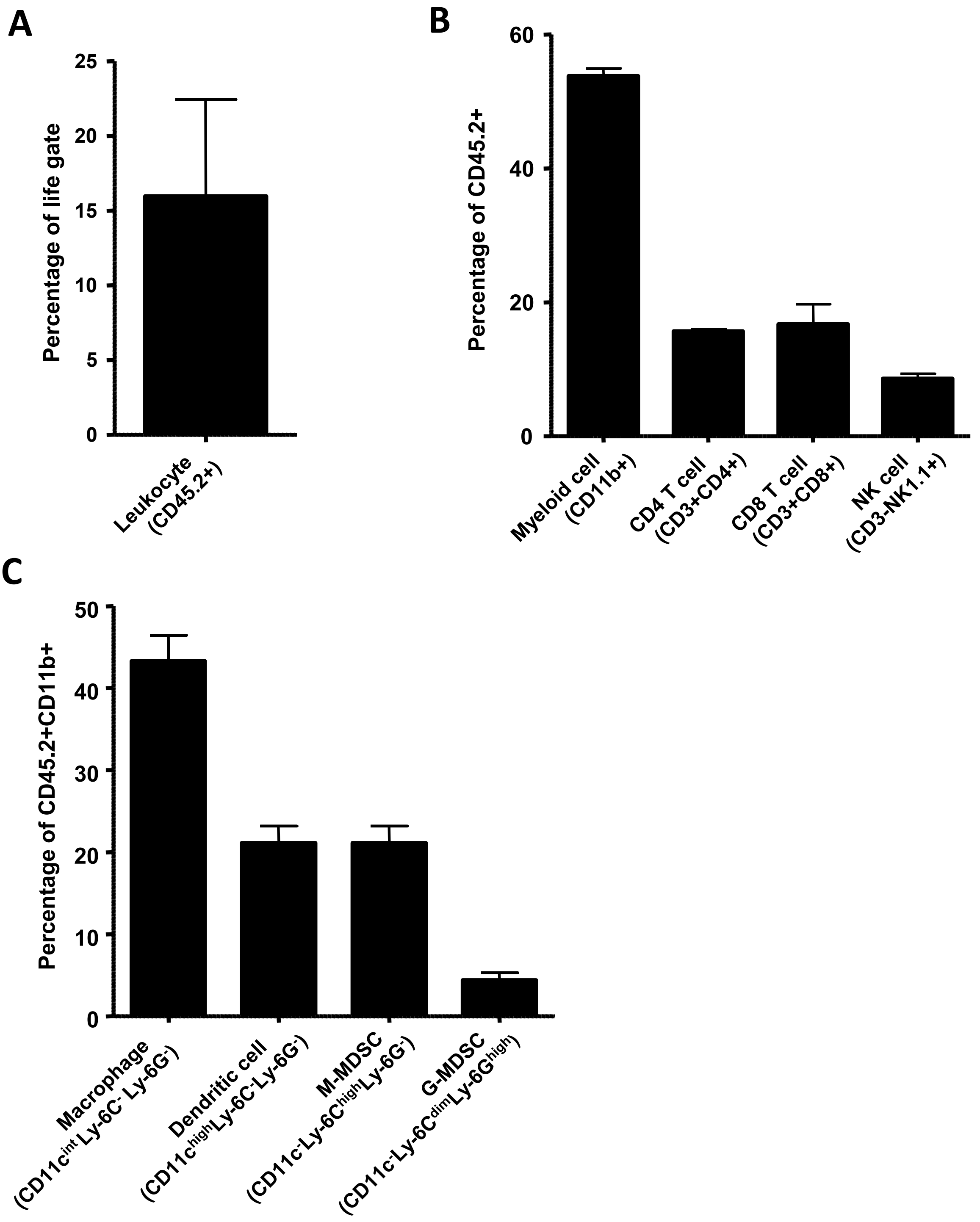

Supplementary Figure 2: Immune infiltration of 9464D-luc tumors. (A) Percentages of tumor infiltrating leukocytes (TIL) in intra-adrenal (IA) 9464D-luc tumors. Total tumor cells in suspension were analyzed for the presence of CD45.2+ TIL. Percentages of CD45.2+ TIL of life gated cells are depicted. (B) Distribution of immune cell types within IA 9464D-luc tumors. CD45.2+ TIL were gated and analyzed for the expression of CD11b, CD3, CD4, CD8 and NK1.1. Percentages of CD11b+ myeloid cells, CD3+CD4+ & CD3+CD8+ T cells and CD3-NK1.1+ NK cells are depicted. (C) The distribution of myeloid cell subsets within total CD45.2 +CD11b+ myeloid cells in IA 9464D-luc tumors. CD45.2+CD11b+ tumor infiltrating myeloid cells were gated and analyzed for the expression of CD11c, Ly-6C, Ly-6G and MHCII. Percentages of CD11c^{int}Ly-6C^{-}Ly-6G^{-} macrophages, CD11c^{high}Ly-6C^{-}Ly-6G^{-} dendritic cells, CD11c^{-} Ly-6C^{high}Ly-6G^{-} M-MDSC and CD11c^{-}Ly- 6C^{dim}Ly-6G^{high} G-MDSC are depicted.
